# Continuum of care for maternal and child health and child undernutrition in Angola

**DOI:** 10.1186/s12889-024-18144-2

**Published:** 2024-03-04

**Authors:** Akiko Saito, Masahide Kondo

**Affiliations:** Department of Health Care Policy and Health Economics, Institute of Medicine, University of Tsukuba, 1-1-1 Tennodai, Tsukuba, Ibaraki 3058577 Japan

**Keywords:** Child nutrition, Undernutrition, Stunting, Underweight, Maternal and child health, Continuum of care, Angola, Africa, Lower middle-income country

## Abstract

**Background:**

Continuum of care (CoC) for maternal and child health provides opportunities for mothers and children to improve their nutritional status, but many children remain undernourished in Angola. This study aimed to assess the achievement level of CoC and examine the association between the CoC achievement level and child nutritional status.

**Methods:**

We used nationally representative data from the Angola 2015–2016 Multiple Indicator and Health Survey. Completion of CoC was defined as achieving at least four antenatal care visits (4 + ANC), delivery with a skilled birth attendant (SBA), child vaccination at birth, child postnatal check within 2 months (PNC), and a series of child vaccinations at 2, 4, 6, 9 and 15 months of child age. We included under 5 years old children who were eligible for child vaccination questionnaires and their mothers. The difference in CoC achievement level among different nutritional status were presented using the Kaplan-Meier method and examined using the Log-Lank test. Additionally, the multivariable logistic regression analysis examined the associations between child nutritional status and CoC achievement levels.

**Results:**

The prevalence of child stunting, underweight and wasting was 48.3%, 23.2% and 5.9% respectively. The overall CoC completion level was 1.2%. The level of achieving CoC of mother-child pairs was 62.8% for 4 + ANC, 42.2% for SBA, 23.0% for child vaccination at birth, and 6.7% for PNC, and it continued to decline over 15 months. The Log-Lank test showed that there were significant differences in the CoC achievement level between children with no stunting and those with stunting (*p* < 0.001), those with no underweight and those with underweight (*p* < 0.001), those with no wasting and those with wasting (*p* = 0.003), and those with malnutrition and those with a normal nutritional status (*p* < 0.001). Achieving 4 + ANC (CoC1), 4 + ANC and SBA (CoC 2), and 4 + ANC, SBA, and child vaccination at birth (CoC 3) were associated with reduction in child stunting and underweight.

**Conclusions:**

The completion of CoC is low in Angola and many children miss their opportunity of nutritional intervention. According to our result, improving care utilization and its continuity could improve child nutritional status.

## Background

Undernutrition affects not only health [1–9] and development [1, 10–15], but also educational attainment [16–18] and productivity [3, 6, 19], in children in the short and long term. Undernourished children are more vulnerable to infections (e.g., those that cause diarrhea and pneumonia) [5] and have a higher risk of morbidity and mortality [2, 5, 7–9]. Undernourished children are also likely to drop out from school more frequently than well-nourished children [6, 19]. Keats EC, et al. (2021) updated 10 core interventions recommended by the 2013 Lancet series [20] and they presented a new framework with 10 direct health-care sector nutritional interventions; maternal and child micronutrient supplementation, maternal and child food supplementation, support for early immediate breastfeeding initiation, delayed cord clamping, promotion and support for exclusive and continued breastfeeding, promotion of age-appropriate complementary feeding practices, management of moderate acute malnutrition, treatment of severe acute malnutrition, anemia treatment, promotion of healthy diet and physical activity during childhood and adolescence [21]. However, the use of these care is an issue, and many children remain undernourished.

In Angola, 38% of children aged younger than 5 years show stunted growth and 19% are underweight [22]. Regarding utilization of maternal and child health (MCH) services, 61% achieve a minimum of four antenatal care visits [22]. The assessment of nutrition is expected to be conducted while women and children visit a health facility for ANC, delivery, child consultations and vaccinations. However, many children miss these opportunities because the coverage of all age-appropriate vaccines in children aged 24–35 months is only 9% [22]. Utilization of health services is hampered by rural residence, a long distance from health facilities, low literacy and education background of mothers, and younger age of mothers in Angola [23]. Other factors are the experience of miscarriage [24], parity [25], women’s and/or household’s wealth [24, 26–29], women’s or parents’ education [23, 28, 30, 31], birth plan [24, 28], ethnicity [28, 30], and sex of the healthcare providers [27] as reported from African countries. However, mothers who use ANC services are more likely to re-visit to receive care [26, 27, 32]. This finding implies women’s continuous visits to MCH services increase the chance of children to receive consultations/vaccinations. Therefore, children have more opportunities to be screened for malnutrition.

Studies that have investigated the situation of continuum of care (CoC) from pregnancy to child vaccinations are limited [33], although an improvement in CoC has been advocated [34]. Seidu A. et al. (2022) reported the Continuum of Care (CoC) level in Angola, which included child vaccinations; however, it also encompassed other cares, such as contraceptive use, and the CoC level was not disaggregated for each care. Furthermore, studies that have examined association between CoC and child undernutrition are scarce [35]. Kuhnt J and Vollmer S (2017) presented negative association between 4 + ANC and child undernutrition, stunting and underweight [35], however, association between child nutrition and CoC which includes child vaccinations are not well known. Therefore, this study aimed to assess the achievement level of CoC for MCH services and examine the association between child nutritional status and the CoC achievement level in Angola.

## Methods

### Data source

The data were derived from the latest Angola 2015–2016 Multiple Indicator and Health Survey (Inquérito de Indicadores Múltiplos e de Saúde em Angola 2015-16; Angola 2015-16 IIMS) dataset which is publicly available [22]. The Angola 2015-16 IIMS is a first nationally representative household survey which consisted of female, male, and child questionnaires. The female questionnaire includes reproductive health data of women aged 15–49 years, and the child questionnaire includes vaccination and nutritional data of children aged 0–59 months.

### Sampling and participants

**Fig. 1:**
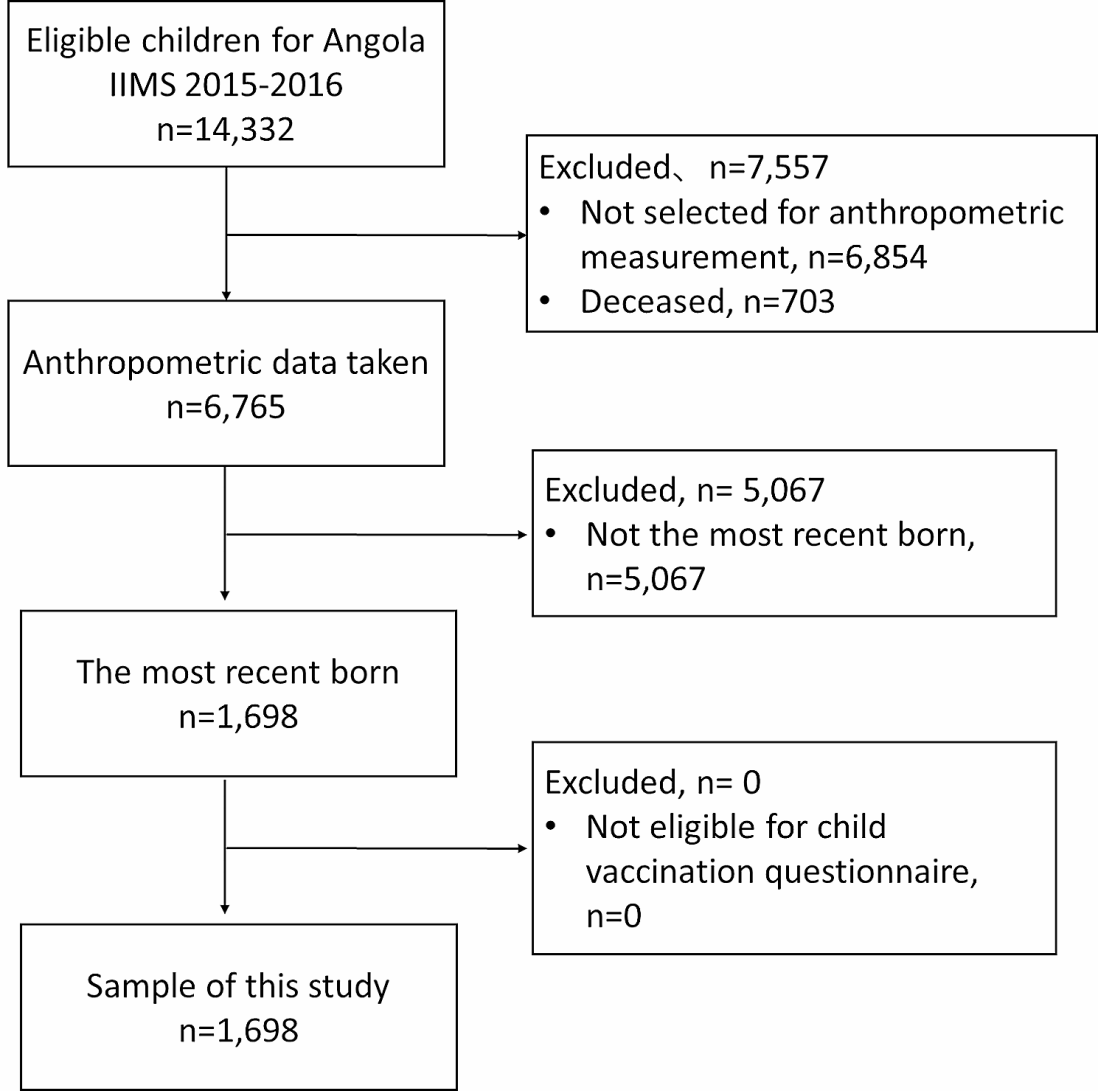
Sampling procedure

Sampling procedure is described in Fig. 1. Detailed sampling strategy of Angola IIMS 2015-16 is described in the IIMS 2015–2016 report [22]. The sample was stratified and selected in three stages. At first stage, 3600 primary sampling units (PSUs) were systematically selected based on census area with probability proportional to size of households in each PSU, from each of 36 stratum which consisted of 18 urban and 18 rural areas from 18 provinces of Angola. Then 900 PSU sub-sample was selected with equal probability within the stratum. At second stage, 627 secondary sampling units (SSUs), of which 345 belong to urban areas and 282 to rural areas were selected within each PSU sub-samples with proportional probability to size. Each SSUs consisted of at least 30 households. Within each selected SSUs, a list of households was made. Finally, 26 households from each SSUs were selected with equal probabilities within the SSUs, resulted in total of 16,302 nationally representative household samples. Then 50% of the selected households were chosen for child anthropometric measurement. Women aged from 15 to 49 years and their children aged from 0 to 59 months who slept in the selected households the previous night were eligible for the Angola IIMS 2015-16 survey. 16,224 households completed the survey (99.2%). Within the selected households, 14,975 (100%) women were eligible for the survey, 14,379 (96%) were interviewed, and 596 (4%) were excluded owing to a lack of consent or no availability for the interview. A total of 14,322 (100%) children born to the 14,379 women were eligible for the survey, and 6765 (47.2%) children with anthropometric measurement data were chosen. Additionally, 7557 (52.8%) children were excluded owing to a lack of anthropometric data (47.9%) or because they were deceased (4.9%). Children born after January 2012 were eligible for child vaccination questionnaires, and both these children and their mothers were included in our analysis. This inclusion was necessary as we aimed to investigate the achievement level of continuous care, assessing all series of child vaccinations. The ANC questions were specifically directed to the most recently born children. Therefore, the final sample size for this study was 1698.

### Nutrition variables

The study variables are described in Table 1. We used the four binary child nutrition variables of stunting (yes = 1, no = 0), underweight (yes = 1, no = 0), wasting (yes = 1, no = 0) and a normal nutritional status (yes = 0, no/malnutrition = 1). The children’s nutritional status was defined by World Health Organization Growth Standards [36]. Stunting was defined by a height-for-age z-score < − 2, underweight was defined by a weight-for-age z-score < − 2, and wasting was defined by a weight-for-height z-score < − 2. Normal nutritional status is defined by not stunted, not underweight, not wasted and not overweight/obese. Overweight/obesity was defined by a weight-for-age z-score > 2.

**Table 1:** Description and categorization of variables used in the logistic regression analysis

Variable name	Description
Stunting	Yes/stunting (a height-for-age z-score < − 2) = 1 No/not stunting (a height-for-age z-score − 2 and above) = 0
Underweight	Yes/ underweight (a weight-for-age z-score < − 2) = 1 No/not underweight (a weight-for-age z-score − 2 and above) = 0
Wasting	Yes/wasting (a weight-for-height z-score < − 2) = 1 No/no wasting (a weight-for-height z-score − 2 and above) = 0
Normal nutritional status	Yes/Normal nutritional status/not stunted, not underweight, not wasted and not overweight and obese (a weight-for-age 2 and less) = 0 No/ malnutrition/ either stunted, underweight, wasted, or overweight/obese (a weight-for-age > 2) = 1
CoC1 achievement level	Four or more antenatal care visits with at least one antenatal care provided by a skilled attendant (doctor, nurse, or birth attendant) Achieved = 1, Not achieved = 0, Missing = 0^1)^
CoC2 achievement level	CoC 1 and child delivery assisted by skilled attendant (doctor, nurse, or birth attendant) Achieved = 1, Not achieved = 0, Missing = 0^1)^
CoC3 achievement level	CoC 2 and child vaccination at birth (Hepatitis B, Polio, and BCG) Achieved = 1, Not achieved = 0, Missing = 0^1)^
CoC4 achievement level	CoC 3 and child’s postnatal care (child’s health checked within 2 months) Achieved = 1, Not achieved = 0, Missing = 0^1)^
CoC5 achievement level	CoC 4 and child vaccination at 2 months of age (DTP, Hib and Hepatitis B or pentavalent, and pneumococcal, polio and rotavirus) Achieved = 1, Not achieved = 0, Missing = 0^1)^
CoC6 achievement level	CoC 5 and child vaccination at 4 months of age (DTP, Hib and Hepatitis B or pentavalent, and pneumococcal, polio, rotavirus, and Vitamin A) Achieved = 1, Not achieved = 0, Missing = 0^1)^
CoC7 achievement level	CoC 6 and child vaccination at 6 months of age (DTP, Hib and Hepatitis B or pentavalent, and pneumococcal and poli0) Achieved = 1, Not achieved = 0, Missing = 0^1)^
CoC8 achievement level	CoC 7 and child vaccination at 9 months of age (Measles/Rubella, Vitamin A and Yellow fever) Achieved = 1, Not achieved = 0, Missing = 0^1)^
CoC9 achievement level	CoC 8 and child vaccination at 15 months of age (Measles/Rubella) Achieved = 1, Not achieved = 0, Missing = 0^1)^
Wealth quintile	Poorest = 1, Poor = 2, Mild = 3, Rich = 4, Richest = 5
Child’s age (months)	15–24 = 1, 25–48 = 2
Sex of child	Boy = 1, Girl = 2
Birth order	1st born = 1, 2nd born = 2, 3rd born = 3, 4th born = 4, 5th born = 5

CoC; continuum of care

1) Missing is categorized as “censoring” in Kaplan-Meier analysis

### Continuum of care variables

In this study, the completion of CoC was defined by four or more ANC visits (at least one ANC visit with a skilled healthcare provider) (4 + ANC) (a), delivery assisted by a skilled birth attendant (SBA) (b), child vaccination at birth (Polio, BCG, Hepatitis B) (c), and child postnatal check within 2 months (postnatal care; PNC) before discharge (d) and at 2 (DTP, Hib and Hepatitis B or pentavalent, and pneumococcal, polio and rotavirus) (e), 4 (DTP, Hib and Hepatitis B or pentavalent, and pneumococcal, polio, rotavirus and Vitamin A) (f), 6 (DTP, Hib and Hepatitis B or pentavalent, and pneumococcal and polio) (g), 9 (Measles/Rubella, Yellow Fever, Vitamin A) (h), and 15 months of age (Measles/Rubella) (i). Child vaccination at birth, 2, 4, 6, 9 and 15 months of age was defined in accordance with Angola vaccination schedule [37]. In Angola IIMS 2015–2016, there are four PNC related variables; “Child’s health checked before discharge”,” Respondent’s health checked before discharge”, “Baby postnatal check within 2 months” and “Respondent’s health checked after discharge/delivery at home”. In this study, we only include “Baby postnatal check within 2 months” because our study included delivery with a skilled birth attendant as CoC2, not institutional delivery, and focused on child outcome. Furthermore, “Respondent’s health checked after discharge/delivery at home” contained many missing values which may affect precision of the analysis. In the generation of variables, a doctor, nurse, or birth attendant was included as a skilled healthcare provider and SBA. We generated the variables CoC 1–9, which were different levels of CoC achievement. CoC 1 was (a), CoC 2 was (a, b), CoC 3 was (a–c), CoC 4 was (a–d), CoC 5 was (a–e), CoC 6 was (a–f), CoC 7 was (a–g), CoC 8 was (a–h), and CoC 9 was (a–i). We did not allow any return to care after dropout since we focused on the continuity of care. For example, if a child skipped 2nd month’s vaccination but returned for 4th month’s vaccination, this child was categorized as CoC not achieved. All CoC variables were coded as achieved = 1 and not achieved = 0. Handling of missing values were different among analysis. In a descriptive analysis and logistic regression analysis, we coded each CoC achieved as “1” and other (not achieved and missing) as “0”. In the Kaplan-Meier method, each CoC achieved was coded “1”, not achieved was coded “0”, and missing values were handled as a “censoring”. Demographic and Health Survey (DHS) Guide defines a missing value as a variable that should have a response but does not, either because the question was not asked (due to interviewer error) or the respondent chose not to answer [38]. Including missing values in the analysis is reasonable, as the care utilization questions may be sensitive, particularly for mothers who have not utilized such care and they may refuse to respond.

### Statistical analysis

Firstly, the CoC achievement level (CoC 1–9) was described as the number and percentage at each level. Secondly, differences in the CoC achievement level between different nutritional statuses were examined by the generalized Wilcoxon test. Finally, the association between the CoC level and child nutrition was identified by a multivariable logistic regression analysis. All analysis was conducted using IBM SPSS 29.0 (IBM Corp., Armonk, NY, USA). **SPSS version 29 complex samples package was used to account for the sampling design.** Individual sample weight, sample strata for sampling errors/design, and cluster number were incorporated in descriptive and logistic regression analysis [38]. Kaplan-Meier method and the Log-Lank test were done without the weight due to technical restriction.

Kaplan-Meier method was applied to draw survival curves to display differences in the CoC achievement level between different nutritional statuses. Log-Lank test was performed to examine their statistical difference. In this analysis, outcome variables were nutritional status; stunting and not stunting, underweight and not underweight, wasting and no wasting, and at normal nutritional status and malnutrition. Event was defined termination of CoC. Missing data was considered “lost follow-up” and handled as censoring. Y-axis displayed proportion of each CoC achieved and X-axis presented CoC 1–9 achievement level. A p-value of < 0.05 were considered statically significant.

Univariable logistic regression analysis was performed to determine the association between child nutritional status and CoC achievement levels and each independent variable. Nine multivariable logistic regression models were estimated for each outcome. Each model was adjusted by covariates; child age (15–23 months and 24–59 months), child’s sex (girl or boy), birth order, and wealth index (poorest, poor, middle, richer, and richest).

Each model contained one of CoC 1 to CoC 9 and all covariates described above. For example, CoC 1 model contained CoC 1 and all covariates described above. Odds ratio (Odds) and adjusted odds ratios (AOR), along with 95% confidence intervals (CI) were calculated. A p-value of < 0.05 were considered statically significant.

## Results

### Sample characteristics

The characteristics of the mothers and children are shown in Table 2. We used data of 1968 mother-child pairs from the Angola 2015-16 IIMS dataset who were chosen for anthropometric measurements using the Angola 2015-16 IIMS sampling strategy and eligible for child vaccination questionnaires. The prevalence of child stunting and underweight was 48.3% and 23.2%, respectively. The prevalence of wasting was 5.9% and that of overweight was 3.6%, and 46.3% of children had a normal nutritional status.

**Table 2:** Summary of sample characteristics (*N* = 1,698, Weighted *N* = 1,580)

	n	%	Weighted n	Weighted %
**Child’s age (months)**				
15–24			865	54.7
25–48			715	45.3
Missing			0	0.0
**Sex of child**				
Boy			774	49.0
Girl			806	51.0
Missing			0	0.0
**Birth order**				
1st born			335	21.2
2nd born			305	19.3
3rd born			249	15.8
4th born			206	13.0
5th born			485	30.7
Missing			0	0.0
**Wealth quintile**				
Poorest			314	19.9
Poor			338	21.4
Mild			349	22.1
Rich			317	20.1
Richest			262	16.6
Missing			0	0.0
**Stunting**				
Yes	762	46.5	728	48.3
No	877	53.5	779	51.7
Missing	59		73	
**Underweight**				
Yes	371	22.6	350	23.2
No	1269	77.4	1161	76.8
Missing	58		69	
**Wasting**				
Yes	91	5.5	89	5.9
No	1562	94.5	1428	94.1
Missing	45		63	
**Overweight**				
Yes	78	4.7	54	3.6
No	1575	95.3	1463	96.4
Missing	45		63	
**Normal**				
Yes	769	46.8	698	46.3
No	873	53.2	811	53.7
Missing	56		71	

n; number of subjects

### CoC achievement level

The CoC achievement levels are shown in Table 3. The overall CoC completion (CoC 9) rate was 1.2%. A total of 62.8% of pregnant women achieved 4 + ANC (CoC 1) and 42.2% had 4 + ANC and delivery assisted by an SBA (CoC 2). The CoC achievement level decreased from 42.2% at CoC 2 to 23.0% at CoC 3 (PNC). A total of 6.7% of pregnant women achieved CoC 4 (child vaccination at birth), 3.1% achieved CoC 5 (at 2 months), 2.1% achieved CoC 6 (at 4 months), and 1.6% achieved CoC 7 (at 6 months), 1.5% achieved CoC 8 (at 9 months), and 1.2% achieved CoC 9 (at 15 months).

**Table 3:** Continuum of care achievement level (Weighted *N* = 1,580)

	Weighted n	Weighted %
CoC 1 achieved	992	62.8
CoC 2 achieved	667	42.2
CoC 3 achieved	364	23.0
CoC 4 achieved	106	6.7
CoC 5 achieved	49	3.1
CoC 6 achieved	34	2.1
CoC 7 achieved	25	1.6
CoC 8 achieved	24	1.5
CoC 9 achieved	20	1.2

n; number of subjects. CoC; continuum of care. CoC 1 was defined as four or more antenatal care visits with at least one ANC visit with a skilled healthcare provider. CoC 2 was defined as CoC 1 and delivery assisted by a skilled birth attendant. CoC 3 was defined as CoC 2 and child vaccination at a birth. CoC 4 was defined as CoC 3 and child postnatal care. CoC 5 was defined as CoC 4 and child vaccination at the 2nd month of childbirth. CoC 6 was defined as CoC 5 and child vaccination at the 4th month of childbirth. CoC 7 was defined as CoC 6 and child vaccination at the 6th month of childbirth. CoC 8 was defined as CoC7 and child vaccination at the 9th month of childbirth. CoC 9 was defined as CoC 8 and child vaccination at the 15th month of childbirth

### Differences in CoC achievement levels by the nutritional status

**Fig. 2:**
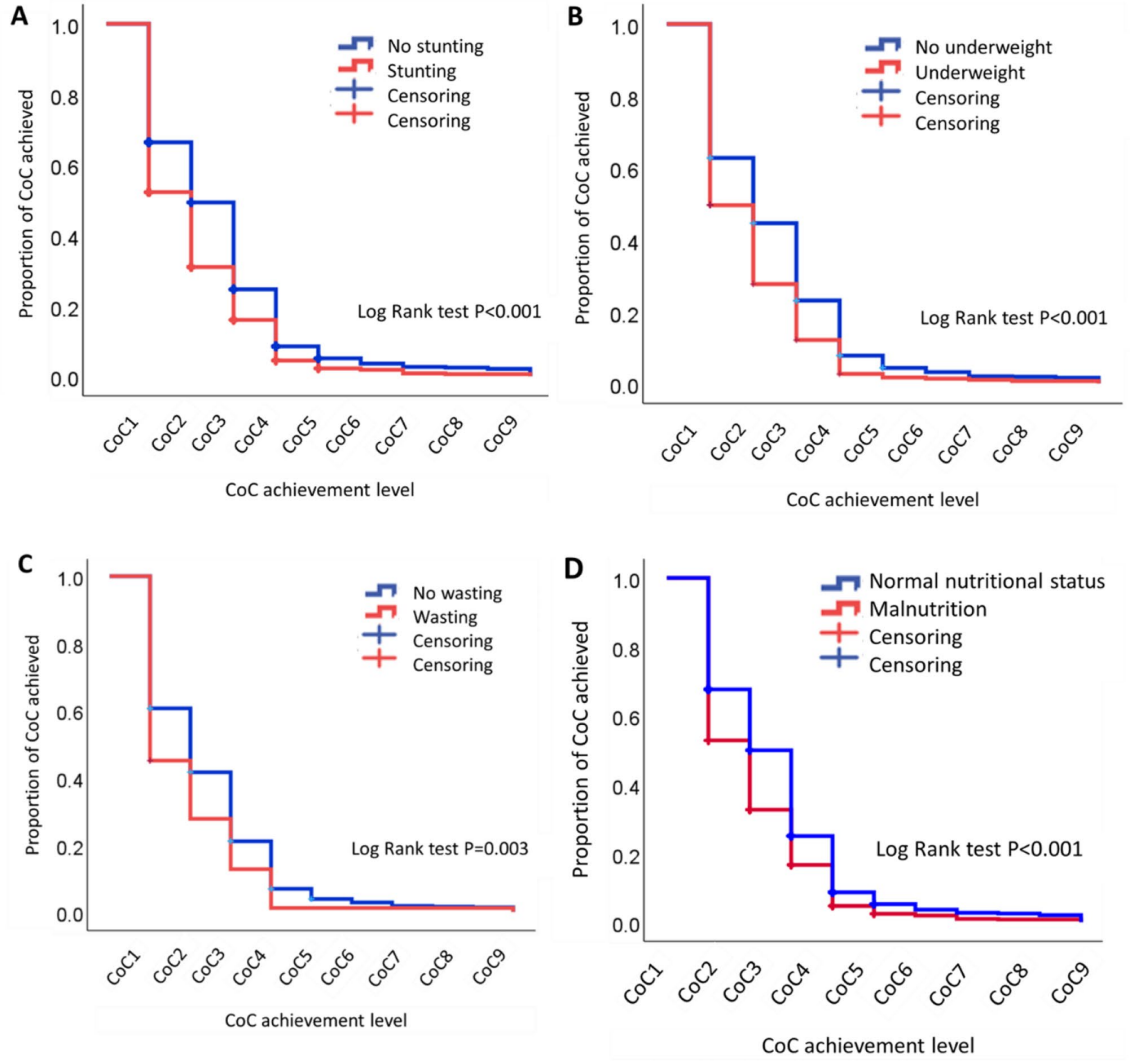
Differences in CoC achievement levels by the nutritional status as shown by Kaplan–Meier curve analysis. (**A**) Stunting vs. no stunting. (**B**) Underweight vs. no underweight. (**C**) Wasting vs. no wasting. (**D**) Normal nutritional status vs. malnutrition. CoC; continuum of care. CoC 1 was defined as four or more antenatal care visits with at least one ANC visit with a skilled healthcare provider. CoC 2 was defined as CoC 1 and delivery assisted by a skilled birth attendant. CoC 3 was defined as CoC 2 and child vaccination at birth. CoC 4 was defined as CoC 3 and child postnatal check within 2 months. CoC 5 was defined as CoC 4 and child vaccination at the 2nd month of childbirth. CoC 6 was defined as CoC 5 and child vaccination at the 4th month of childbirth. CoC 7 was defined as CoC 6 and child vaccination at the 6th month of childbirth. CoC 8 was defined as CoC7 and child vaccination at the 9th month of childbirth. CoC 9 was defined as CoC 8 and child vaccination at the 15th month of childbirth

Kaplan-Meier curves illustrating differences in CoC achievement levels based on the nutritional status are shown in Fig. 2, while the CoC achievement level based on the nutritional status are shown in Table 4. Kaplan–Meier curve was drawn, and the Log-Lank test compared difference among different nutritional status. The Kaplan–Meier curves showed that children with no stunting, those who were not underweight and did not have wasting, and those with a normal nutritional status had a higher CoC achievement level than children with stunting, underweight, wasting, or malnutrition. The Log-Lank test showed significant differences in CoC achievement levels between children with no stunting and those with stunting (*p* < 0.001), those with no underweight and those with underweight (*p* < 0.001), those with no wasting and those with wasting (*p* = 0.003), and those with malnutrition and those with a normal nutritional status (*p* < 0.001).

**Table 4:** Continuum of care achievement levels by nutritional status

	Stunting				Underweight				Wasting				Normal nutritional status			
	Yes		No		Yes		No		Yes		No		Yes		No	
	n	%	n	%	n	%	n	%	n	%	n	%	n	%	n	%
**CoC1 completion**																
Achieved	381	50.0	564	64.3	174	46.9	771	60.8	39	42.9	914	58.5	505	65.7	441	50.5
Not achieved	375	49.2	304	34.7	195	52.6	485	38.2	52	57.1	633	40.5	255	33.2	426	48.8
Censoring	6	0.8	9	1.0	2	0.5	13	1.0	0	0.0	15	1.0	9	1.2	6	0.7
**CoC2 completion**																
Achieved	219	28.7	416	47.4	96	25.9	539	42.5	24	26.4	617	39.5	371	48.2	265	30.4
Not achieved	537	70.5	452	51.5	273	73.6	717	56.5	67	73.6	930	59.5	389	50.6	602	69.0
Censoring	6	0.8	9	1.0	2	0.5	13	1.0	0	0.0	15	1.0	9	1.2	6	0.7
**CoC3 completion**																
Achieved	108	14.2	198	22.6	41	11.1	265	20.9	11	12.1	297	19.0	176	22.9	130	14.9
Not achieved	640	84.0	664	75.7	325	87.6	980	77.2	79	86.8	1237	79.2	579	75.3	728	83.4
Censoring	14	1.8	15	1.7	5	1.3	24	1.9	1	1.1	28	1.8	14	1.8	15	1.7
**CoC4 completion**																
Achieved	26	3.4	50	5.7	8	2.2	68	5.4	1	1.1	75	4.8	43	5.6	33	3.8
Not achieved	719	94.4	805	91.8	356	96.0	1169	92.1	89	97.8	1449	92.8	705	91.7	822	94.2
Censoring	17	2.2	22	2.5	7	1.9	32	2.5	1	1.1	38	2.4	21	2.7	18	2.1
**CoC5 completion**																
Achieved	11	1.4	27	3.1	5	1.3	33	2.6	1	1.1	37	2.4	23	3.0	15	1.7
Not achieved	716	94.0	803	91.6	353	95.1	1167	92.0	88	96.7	1445	92.5	703	91.4	819	93.8
Censoring	35	4.6	47	5.4	13	3.5	69	5.4	2	2.2	80	5.1	43	5.6	39	4.5
**CoC6 completion**																
Achieved	9	1.2	19	2.2	4	1.1	24	1.9	1	1.1	27	1.7	16	2.1	12	1.4
Not achieved	718	94.2	811	92.5	354	95.4	1176	92.7	88	96.7	1455	93.1	710	92.3	822	94.2
Censoring	35	4.6	47	5.4	13	3.5	69	5.4	2	2.2	80	5.1	43	5.6	39	4.5
**CoC7 completion**																
Achieved	4	0.5	14	1.6	3	0.8	15	1.2	1	1.1	17	1.1	12	1.6	6	0.7
Not achieved	723	94.9	816	93.0	355	95.7	1185	93.4	88	96.7	1465	93.8	714	92.8	828	94.8
Censoring	35	4.6	47	5.4	13	3.5	69	5.4	2	2.2	80	5.1	43	5.6	39	4.5
**CoC8 completion**																
Achieved	3	0.4	13	1.5	2	0.5	14	1.1	1	1.1	15	1.0	11	1.4	5	0.6
Not achieved	724	95.0	812	92.6	356	96.0	1181	93.1	88	96.7	1462	93.6	710	92.3	829	95.0
Censoring	35	4.6	52	5.9	13	3.5	74	5.8	2	2.2	85	5.4	48	6.2	39	4.5
**CoC9 completion**																
Achieved	3	0.4	11	1.3	2	0.5	12	0.9	1	1.1	13	0.8	9	1.2	5	0.6
Not achieved	724	95.0	814	92.8	356	96.0	1183	93.2	88	96.7	1464	93.7	712	92.6	829	95.0
Censoring	35	4.6	52	5.9	13	3.5	74	5.8	2	2.2	85	5.4	48	6.2	39	4.5

CoC; continuum of care. CoC 1 was defined as four or more antenatal care visits with at least one ANC visit with a skilled healthcare provider. CoC 2 was defined as CoC 1 and delivery assisted by a skilled birth attendant. CoC 3 was defined as CoC 2 and child vaccination at a birth. CoC 4 was defined as CoC 3 and child postnatal care. CoC 5 was defined as CoC 4 and child vaccination at the 2nd month of childbirth. CoC 6 was defined as CoC 5 and child vaccination at the 4th month of childbirth. CoC 7 was defined as CoC 6 and child vaccination at the 6th month of childbirth. CoC 8 was defined as CoC7 and child vaccination at the 9th month of childbirth. CoC 9 was defined as CoC 8 and child vaccination at the 15th month of childbirth

### Association between children’s nutritional status and CoC achievement levels

The result of the logistic regression analysis is shown in Table 5. Achieving CoC 1 (AOR: 0.60, 95% CI: 0.443–0.821), CoC 2 (AOR: 0.39, 95% CI: 0.285–0.535), and CoC 3 (AOR: 0.42, 95% CI: 0.284–0.617), had smaller odds of being stunted compared to not achieving each CoC. Achieving CoC 1 (AOR: 0.68, 95%CI: 0.489–0.939), CoC 2 (AOR: 0.53 0.70, 95% CI: 0.329 0.533–0.845 0.911) and CoC 3 (AOR: 0.40 0.70, 95% CI: 0.239 0.496–0.675 0.968) had smaller odds of being underweight compared to not achieving each CoC. No significant association between wasting and CoC was not observed.

**Table 5:** Result of logistic regression analysis

	Stunting			Underweight			Wasting		
	n (%)	OR (95%CI)	AOR (95%CI)	n (%)	OR (95%CI)	AOR (95%CI)	n (%)	OR (95%CI)	AOR (95%CI)
CoC1 (achieved)	381 (40.3)	0.55 (0.401–0.749)	0.60 (0.443–0.821)	174 (18.4)	0.59 (0.432–0.807)	0.68 (0.489–0.939)	39 (4.1)	0.61 (0.369–1.015)	0.65 (0.389–1.086)
CoC2 (achieved)	219 (34.5)	0.36 (0.262–0.481)	0.39 (0.285–0.535)	96 (15.1)	0.44 (0.29–0.679)	0.53 (0.329–0.845)	67 (6.7)	0.76 (0.4-1.451)	0.86 (0.45–1.623)
CoC3 (achieved)	108 (35.3)	0.38 (0.257–0.559)	0.42 (0.284–0.617)	41 (13.4)	0.34 (0.208–0.561)	0.402 (0.239–0.675)	11 (3.6)	0.50 (0.193–1.294)	0.54 (0.211–1.382)
CoC4 (achieved)	26 (34.2)	0.45 (0.201–1.005)	0.48 (0.217–1.066)	8 (10.5)	0.33 (0.106–1.02)	0.39 (0.123–1.211)	1 (1.3)	0.09 (0.012–0.695)	0.20 (0.013–0.717)
CoC5 (achieved)	11 (28.9)	0.32 (0.077–1.364)	0.33 (0.083–1.331)	5 (13.2)	0.37 (0.087–1.613)	0.42 (0.097–1.818)	1 (2.6)	0.21 (0.027–1.649)	0.20 (0.025–1.592)
CoC6 (achieved)	9 (32.1)	0.53 (0.114–2.481)	0.52 (0.116–2.364)	4 (14.3)	0.56 (0.132–2.379)	0.61 (0.139–2.669)	1 (3.6)	0.31 (0.038–2.525)	0.29 (0.035–2.42)
CoC7 (achieved)	4 (22.2	0.52 (0.067–4.009)	0.53 (0.073–3.831)	3 (16.7)	0.77 (0.176–3.401)	0.91 (0.211–3.933)	1 (5.6)	0.43 (0.049–3.73)	0.39 (0.043–3.55)
CoC8 (achieved)	3 (18.8)	0.52 (0.064–4.255)	0.54 (0.071–4.071)	2 (12.5)	0.76 (0.163–3.546)	0.91 (0.2-4.113)	1 (6.3)	0.44 (0.05–3.886)	0.41(0.044–3.746)
CoC9 (achieved)	3 (21.4)	0.73 (0.085–6.281)	0.81 (0.112–5.912)	2 (14.3)	0.99 (0.229–4.297)	1.28 (0.339–4.859)	1 (7.1)	0.55 (0.058–5.103)	0.53 (0.053–5.33)

n; number of stunting, %: percentage of stunting, OR; Odds Ratio. CI; Confidential Interval. AOR; Adjusted Odds Ratio. CoC; continuum of care. CoC 1 was defined as four or more antenatal care visits with at least one ANC visit with a skilled healthcare provider. CoC 2 was defined as CoC 1 and delivery assisted by a skilled birth attendant. CoC 3 was defined as CoC 2 and child vaccination at a birth. CoC 4 was defined as CoC 3 and child postnatal care. CoC 5 was defined as CoC 4 and child vaccination at the 2nd month of childbirth. CoC 6 was defined as CoC 5 and child vaccination at the 4th month of childbirth. CoC 7 was defined as CoC 6 and child vaccination at the 6th month of childbirth. CoC 8 was defined as CoC7 and child vaccination at the 9th month of childbirth. CoC 9 was defined as CoC 8 and child vaccination at the 15th month of childbirth. Multivariable logistic regression models were adjusted with child age, child's sex, birth order and wealth quintile

## Discussion

To the best of our knowledge, this is the first study to assess the achievement level of continuous care utilization by mother-child pairs from pregnancy to 15th month of child vaccination in Angola. There are numbers of studies assessed CoC achievement levels, however, most of them did not include child vaccination [37–47]. Vaccinations prevent children from diseases which may result in child undernutrition. Furthermore, visiting health facility for vaccination also increase children’s opportunity to be screened for nutritional status. Association of CoC with child undernutrition had described previously, however, only ANC was taken into account [36]. Our study analyzed association between CoC from pregnancy to 15th month of child vaccination and undernutrition by Kaplan-Meier method and the Log-Lank test, and logistic regression analysis.

### CoC achievement level

This study showed a low CoC completion level. Only 1.2% of mother-child pairs received continuous care of 4 + ANC, child delivery assisted by an SBA, child vaccination at birth, PNC, and all series of child vaccinations. Comparing our findings with the previous studies from Angola and neighboring countries is difficult because the definition of CoC completion varies among studies. Seidu A. et al. (2022) reported a CoC achievement level of 1.2% in Angola, including 4 + ANC, neonatal tetanus protection, facility-based delivery, Skilled Birth Attendant (SBA), PNC within the first 2 days after birth, BCG, DPT, Polio, Measles, age-appropriate breastfeeding, and current use of modern contraceptives [48]. The CoC completion levels, including all series of child vaccinations, in Myanmar and Timor-Leste were 4.0% and 5.6%, respectively [33]. Angola and these two countries have been affected by war or domestic conflict.

Pregnant women who had 4 + ANC was 62.8%. Therefore, 37.2% of these women did not meet the minimum requirement specified by the World Health Organization of at least four ANC visits [49]. In the same sample, the percentage of pregnant women who had at least one ANC visit was 81.9% (data not shown). Therefore, the utilization of care continuity needs to be strengthened. A long distance to a health facility [50], the mother’s low education level [23, 28, 30, 31], the partner’s low education level [48], less participation of women in decision-making [48], economic restrictions [24, 26–29], and a lack of knowledge regarding ANC [51] prevent mothers from utilizing ANC. A delay in a first ANC visit also contributes to less frequency of ANC visits [52].

There was 20.6% difference between women with 4 + ANC (62.8%) and those with 4 + ANC and child delivery assisted by an SBA (42.2%) in this study. Previous studies show that the utilization [53] and better experience [54] of ANC leads mothers to deliver at a health facility and/or with an SBA and mothers who were not exposed to delivery care information were less likely to deliver at a health facility. Therefore, this low percentage in our study suggested that health care providers did not sufficiently educate mothers on the importance of delivery with an SBA during ANC. There are also other reasons why women avoid delivery with an SBA or institutional delivery, such as economic restrictions [24, 26–29] and a long distance to a health facility [23].

The CoC achievement level decreased by 19.2% from child delivery assisted by an SBA (42.2%) to child vaccination at birth (23.0%). A total of 6.7% of pregnant women achieved CoC 4, which was a 16.3% decrease in CoC achievement level from child vaccination at birth to PNC. Child vaccination at birth should be offered at a health facility before discharge or at the place of birth within 24 h [55]. This situation suggests there is an issue regarding the service provider in that they may not appropriately offer the necessary care.

In this study, only 3.1% of mother-child pairs achieved CoC for child vaccination at 2 months, and this level decreased to 1.2% at 15 months. The achievement level of continuous care regarding child vaccinations is very low and many children miss opportunities to be screened for their nutritional status. Mothers usually bring children to a health facility for vaccinations; therefore, the mothers’ decision to act is important for child vaccination. Oliveira et al. described that being aware of a vaccination program was associated with uptake of child vaccinations in Angola [56]. Therefore, raising awareness before childbirth enables children to receive vaccinations.

### Differences in CoC achievement levels by the nutritional status

The importance of CoC to improve child nutrition has been advocated [34], but supporting evidence is limited. Therefore, this study attempted to examine differences in CoC achievement levels by different nutritional statuses and added new evidence. To the best of our knowledge, this is the first study to report a significant difference in the CoC achievement levels which include child vaccination by child nutritional status. Our study is unique because it analyzed the difference in CoC achievement trends according to child nutritional status by performing the Log-Lank test. We found that children who were not stunted, were not underweight, did not have wasting, or had a normal nutritional status achieved a higher CoC level than children who were stunted, underweight, wasted, or malnourished.

### Association between child undernutrition and CoC achievement levels

The associations between each CoC achievement level and child nutritional status were examined using a multivariable logistic regression analysis. The CoC was partially associated with child stunting and underweight.

Achieving 4 + ANC (CoC 1), 4 + ANC and delivery with a skilled birth attendant (CoC 2), and 4 + ANC, delivery with a skilled birth attendant and child vaccination at birth (CoC 3) were associated with reduction in child stunting and underweight. A positive association between stunting and 4 + ANC as well as underweight and 4 + ANC has previously been described [35]. However, this study is the first to show an association between further continuous care utilization (CoC 2 and CoC 3) and better child nutrition. Significant association between above CoC level 4 (CoC 4–9) and undernutrition were not observed. It is probably due to small number of mother-child pairs who achieved those CoC levels. In addition, there is also a possibility of underserved. Even mother-child pair reached the care, appropriate nutrition service may not be offered. Evidence from rural Kenya showed that approximately 12% of children were not properly assessed or did not have their growth recorded at the time of their vaccinations [57].

### Limitations

There are some limitations to this study. Firstly, restricting participants to children eligible for vaccination questionnaires, along with their mothers, compromised the representativeness of the data. The percentage of stunting in this study was 46.5% (not weighted), while it was reported as 38% in the Angola 2015-16 report [22]. However, to assess the level of care continuity, including from ANC to all series of child vaccinations, which are evidently important for child nutrition, limiting participants could not be avoided. Secondly, a certain number of missing values may have affected the quality of the analysis, even though these values constituted less than 5%. The DHS defines a missing value as a variable that should have a response but does not, either because the question was not asked (due to interviewer error) or the respondent chose not to answer [38]. There is a possibility of non-response bias, especially regarding questions about the utilization of MCH care. These questions might be sensitive, particularly for mothers who have not utilized such care. Lastly, there are disadvantages in using secondary data. The variable of child consultation did not exist, even though nutrition assessment, counselling and other necessary intervention were conducted during child consultations. We also could not guarantee that all ANC were provided by a skilled provider because “at least one ANC visit with a skilled provider” was the only applicable variable. Therefore, because of these limitations, our study may have overestimated or underestimated the CoC achievement level in mothers and children in Angola. Recall bias, especially for older children, also needs to be taken into consideration. Due to the nature of Angola IIMS 2015–2016 methodology, interviewed mothers for their children age of under 5 years, recall bias could not be avoided. Despite these limitations, using the Angola 2015-16 IIMS dataset was the best method to attain the study objectives because it included mothers and children who did not utilize MCH services or dropped out from these services.

## Conclusion

Our study assessed the achievement level of CoC for MCH services and examined the association between the child nutritional status and CoC achievement level in Angola. This study suggests that the CoC completion level is low in Angola. However, among mother-child pairs who achieve a high CoC level, children tend to have no stunting, are not underweight, do not have wasting, and have a normal nutritional status. A low CoC (CoC 1–3) achievement level is associated with undernutrition in children. These findings suggest that early involvement of women in MCH services and education of mothers regarding the importance of utilizing MCH services are required to prevent child malnutrition. Further studies are required to investigate the implementation status of nutritional screening and other nutritional interventions, and the delivery of MCH service information at every visit. According to our result, improving care utilization and its continuity could improve child nutritional status.

## Data Availability

Data are available upon requests made to MEASURE DHS (URL: http://www.dhsprogram.com).

## Abbreviations

CoC: Continuum of Care
ANC: Antenatal care
SBA: Skilled birth attendant
PNC: Postnatal care
MCH: Maternal and child health
IIMS: Multiple Indicator and Health Survey (Inquérito de Indicadores Múltiplos e de Saúde em Angola 2015-16)
DHS: Demographic and Health Survey
OR: Odds ratio
aOR: adjusted odds ratio
95%CI: 95% confidence interval
